# Research trends from 1992 to 2022 of acupuncture anesthesia: a bibliometric analysis

**DOI:** 10.3389/fmed.2023.1194005

**Published:** 2023-06-08

**Authors:** Linxi Sun, Xuqiang Wei, Ke Wang, Jia Zhou

**Affiliations:** ^1^Acupuncture Anesthesia Clinical Research Institute, Yueyang Hospital of Integrated Traditional Chinese and Western Medicine, Shanghai University of Traditional Chinese Medicine, Shanghai, China; ^2^Office of National Clinical Research Base of TCM, Yueyang Hospital of Integrated Traditional Chinese and Western Medicine, Shanghai University of Traditional Chinese Medicine, Shanghai, China

**Keywords:** acupuncture, anesthesia, bibliometric analysis, CiteSpace, VOSviewer

## Abstract

**Background:**

Acupuncture anesthesia is a significant technical development that originated in China in 1958 and was introduced to the West in the early 1970s. Due to its relative novelty, it has been the subject of intense scrutiny and contestation. Since the early 1970s, the use of acupuncture as a complementary treatment for opioid analgesics has been accepted. Research on acupuncture anesthesia has helped to reduce clinical opioid abuse. However, only a few articles have focused on previous publications that reflect the trend of the study, the main investigators, reciprocal collaboration, and other information in this field. In view of this, we utilized bibliographic analysis methods to objectively analyze current trends and research hotspots in this field, aiming to provide a foundation and reference for future studies.

**Methods:**

The Web of Science database was searched for publications related to acupuncture anesthesia between 1992 and 2022. The CiteSpace and VOSviewer were used to analyze the annual publications, authors, Co-cited authors, and their countries (regions) and institutions, co-occurrence keywords, burst keywords, Co-citation references and Co-citation journals.

**Results:**

A total of 746 eligible publications were retrieved from the database for the analysis, including 637 articles and 109 reviews. And the trend of annual publications continued to grow. Aashish J. Kumar, Daniel I. Sessler, Baoguo Wang, and Paul F. White published the most papers in this field (7), and all authors, had a very low centrality (<0.01). China (252) and the University of California System (21) were the most productive country (region) and institution, respectively, while the United States (0.62) and University of California System (0.16) had the highest centrality. After removing keywords related to the search strategy, the three most frequent were pain (115), electroacupuncture (109), and stimulation (91). The six most recent burst keywords were recovery, transcutaneous electrical acupoint stimulation, systematic review, quality, general anesthesia, and surgery. Wang et al.’s article had the highest co-citation count (20), whereas Zhang et al.’s articles had the highest centrality (0.25). The Journal of *Anesthesia and Analgesia* was the most influential one (408 co-citations).

**Conclusion:**

This research provides valuable information for the study of acupuncture anesthesia. In recent years, frontier topics in acupuncture anesthesia research have been the promotion of perioperative rehabilitation, anesthesia management, and quality improvement.

## 1. Background

Acupuncture anesthesia refers to the use of acupuncture to obtain or strengthen anesthesia by following meridians, identifying evidence, or localizing points of acupuncture according to different diseases and surgical sites (1). It is a unique anesthesia method in China that uses acupuncture therapy to assist in surgery. Acupuncture anesthesia is a new exploration of anesthesiology and an original research field in the history of Chinese medicine (2, 3). It perfectly combines the ancient acupuncture technique with the modern anesthesia technique in the surgical field and is a typical case of the integration of traditional Chinese and Western medicine (4).

During President Nixon’s historic visit to China in 1972, members of his delegation observed thyroidectomy and lobectomy surgeries performed under acupuncture anesthesia. This event marked the beginning of a global surge in interest and research into acupuncture anesthesia (5, 6). It is well known that surgery occupies an unshakable position in Western medicine, and traditional Chinese acupuncture can be combined with surgery, which has promoted acupuncture therapy worldwide. Multiple clinical studies demonstrated that combining acupuncture with anesthesia leads to improved patient outcomes, including decreased preoperative anxiety, less stress response during surgery, improved immune function, and fewer postoperative side effects (7–11). The indications for acupuncture anesthesia are also expanding, not only for various surgical procedures (12) but also for gynecological procedures such as abortion (13) and cesarean section (14), as well as local trauma-assisted examinations such as peritoneal dialysis (15) and gastroscopy (16). These advancements have significantly contributed to the improvement of individuals’ overall health.

In recent years, the number of research papers on acupuncture anesthesia published in high-level domestic and international journals has increased rapidly, and acupuncture anesthesia research is progressing in a scientific and standardized manner (17). However, despite enthusiasm in the clinical and research fields (18, 19), no studies conducted to systematically organize and deeply analyze research trends in acupuncture anesthesia, which to some extent restricts the advancement of the general research of acupuncture anesthesia (20). Therefore, an in-depth study of this field using bibliometric analysis is highly warranted.

Bibliometric analysis is a method of evaluating and quantifying information in the literature using mathematical and statistical methods, which helps to gain a complete understanding of research progress in a scientific field (21, 22). This analysis has been applied to several fields of acupuncture, including acupuncture for cardiac disease and postoperative analgesia, and many research results have been accumulated (23–25). The bibliometric approach was applied in this study to analyze the literature on acupuncture anesthesia over the last 31 years from multiple perspectives, such as authors, institutions, countries, keywords, co-cited references, and co-cited journals. And the results were presented in the form of scientific knowledge maps by using the CiteSpace software; then the maps were further interpreted and analyzed to gain an intuitive and comprehensive understanding of the research in the field, identify research hotspots, and provide new research ideas (26, 27).

## 2. Materials and methods

### 2.1. Data sources and search strategy

All data for this study were obtained from the Web of Science (WoS) Core Collection database. Relative to general databases such as Scopus, Derwent, China National Knowledge Infrastructure (CNKI), and the Chinese Social Sciences Citation Index (CSSCI), WOS includes more scientific publications and provides overall data sources for bibliometric software. Thus, WOS is the most frequently used database in bibliometric research (28, 29). The terms “acupuncture” and “anesthesia” were used in the MeSH search.^1^ Data retrieval strategies were established by referring to the Mesh terms tree and related literature for additional information (25, 30, 31). Timespan: 01-01-1992 to 31-12-2022. (Retrieved on January 30, 2023). Seven hundred and eighty-eight documents were obtained. Only the type of article and review document was retained, which were formally published and had comprehensive research data. There were no restrictions on the language or type of research. Duplicate records were removed. Finally 746 documents were included in the analysis. Specific search strategies and results are shown in Table 1.

**Table 1:** The topic search query for web of science.

Set	Results	Search query
#1	28,384	TS = (Acupuncture OR Pharmacopuncture OR Acupressure OR Acupuncture Therapy OR Acupuncture Point∗ OR acupunct* OR needl* OR Electroacupuncture OR Transcutaneous Electrical Acupoint Stimulation OR Ear Acupuncture OR Auricular acupuncture OR Laser Acupuncture OR meridian∗ OR acupoint∗)
#2	207,332	TS = (Anesthesia* OR anesthesia* OR anesthetic* OR “Hypnosis Anesthetic” OR Anaesthetization OR Narcosis* OR Narcotism* OR “Nerve Block” OR Cryoanesthesia)
#3	788	#1 AND #2

### 2.2. Bibliometrics and visualization analysis

We exported retrieved articles in plain text format with full records and references, named “download_XXX.txt” and then imported into CiteSpace (version 6.1.R6 64-bits). CiteSpace combined with Excel was applied for data organization, the centralities calculations, and visual analysis, including: (1) statistical and descriptive analysis: for parameters such as annual publication volume, authors, countries, and institutions; (2) collaborative network analysis: mainly for the three dimensions of countries (regions), institutions and authors; (3) co-occurrence analysis: for keywords; (4) citation burst analysis: mainly for keywords; (5) co-citation analysis: for authors, references and journals.

The VOSviewer (version 1.6.19) was used to optimize and supplement the unaesthetic map. The different nodes represent different items, while the size of the circle, determined by the weight of the item, reflects productivity. The lines between items represent links. Scimago Graphica 1.0.26^2^ was used to visualize country distribution and partnerships. MapEquation^3^ is used to produce a keyword alluvial diagram. Thicker lines mean stronger links and closer cooperation. The impact factor (IF) and the H-index^4^ were added to the table for a comprehensive and scientometric result analysis.

The specific parameters for the visualization analysis were set as follows: The threshold of “Top N% per slice” was 50 for all calculations. The time span was from January 1992 to December 2022, and the time slice setting for all analyzes conducted with CiteSpace was “1 year per slice.” The clustering labels were extracted using the LLR algorithm (21). When mapping visualization knowledge figures, we followed the main procedural steps of CiteSpace, including time slicing, thresholding, modeling, pruning, merging, and mapping (32). Central concepts of CiteSpace include burst detection, betweenness centrality, and heterogeneous networks, which can help to timely visualize the research status, hot spots, and frontiers.

### 2.3. Charts interpretation

CiteSpace generates maps composed of nodes representing the objects under analysis (e.g., authors, institutions, or keywords). The diameter of these nodes corresponds to the frequency of the analyzed objects, for instance, the output or citation frequency. The color of the nodes varies according to the year of publication, and the lines connecting them represent collaborations or co-occurrences. The color of the lines indicates the time of the first collaboration, while the thickness of the lines reflects the strength of the collaboration (33).

## 3. Results

### 3.1. Search results and study characteristics

**Figure 1:**
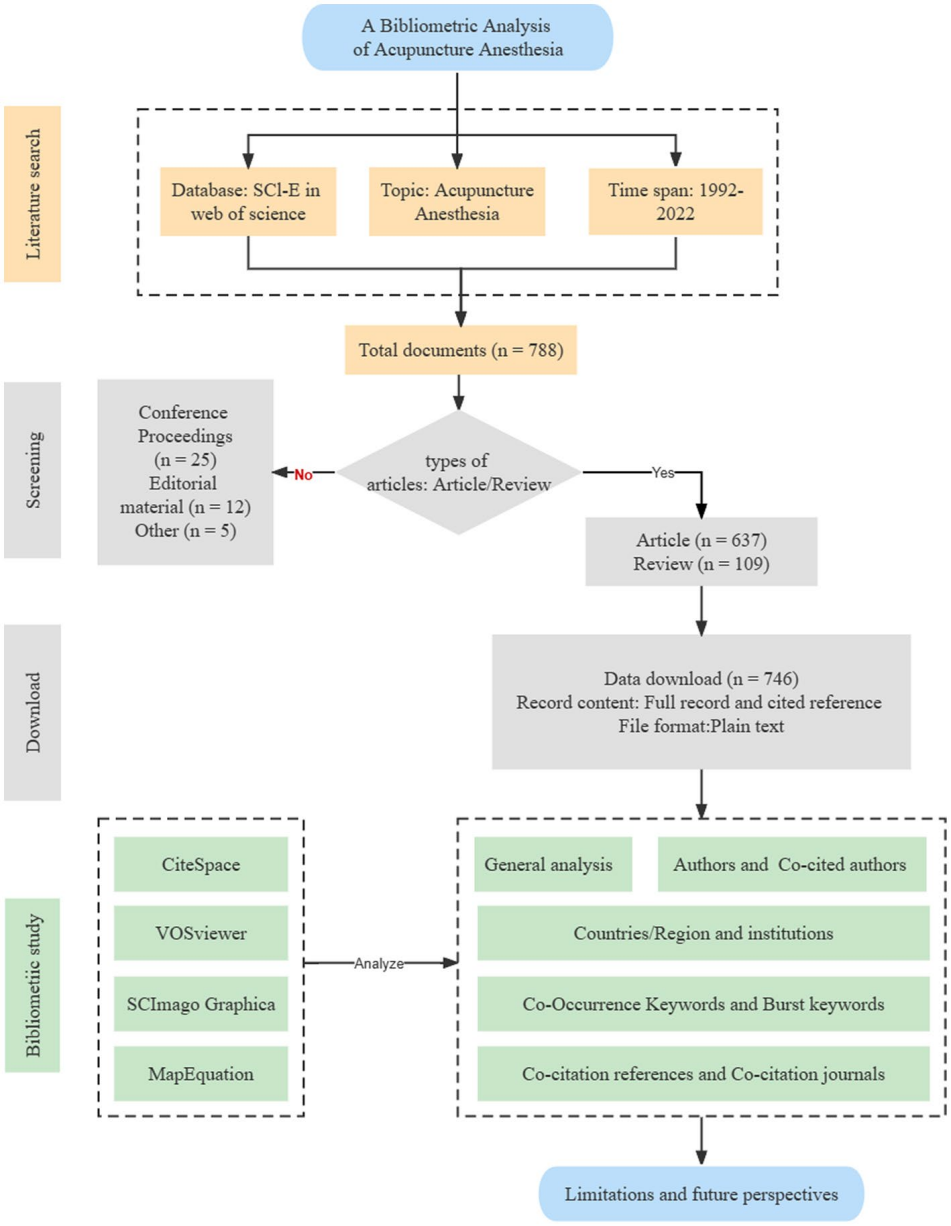
Flow chart indicating the selection process for this bibliometric analysis.

After duplicating removal function in CiteSpace software and manually cleaning the merged data, 746 studies were finally identified. Amid these document types, the Article and the Review had the percent of 85.39 and 14.61%, respectively. A flowchart of the screening process is presented in Figure 1.

### 3.2. Annual publications and trends

**Figure 2:**
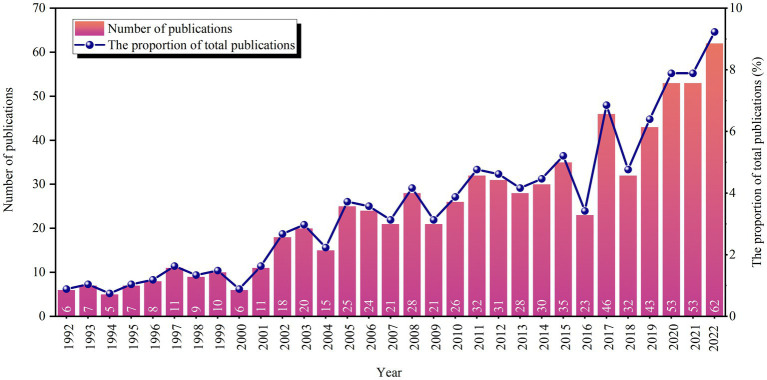
Annual trend of publications.

In the field of acupuncture anesthesia, there has been a steady increase in the number of publications over the past 31 years, with some fluctuations ranging from 5 to 62 publications. Notably, from 2016 to 2017, the number of related publications increased the most with 23 publications. This increase in publication indicates growing interest from researchers in this field. Please refer to Figure 2 for the number and trend of annual articles.

From 2005 to 2016, there was a stable trend in the publication of articles, with an average of around 27 articles per year. However, in 2017, there was an increase in the number of publications to 46. After a temporary decline, the period from 2018 to 2022 saw a sustained and significantly accelerated increase in publication rates. The average number of publications during this period was over 52 per year, which is the highest recorded over the past 31 years.

To facilitate a quick look at representative articles, the top ten cited articles and their features and findings are in Supplementary material.

### 3.3. Analysis of countries/region and institutions

**Figure 3:**
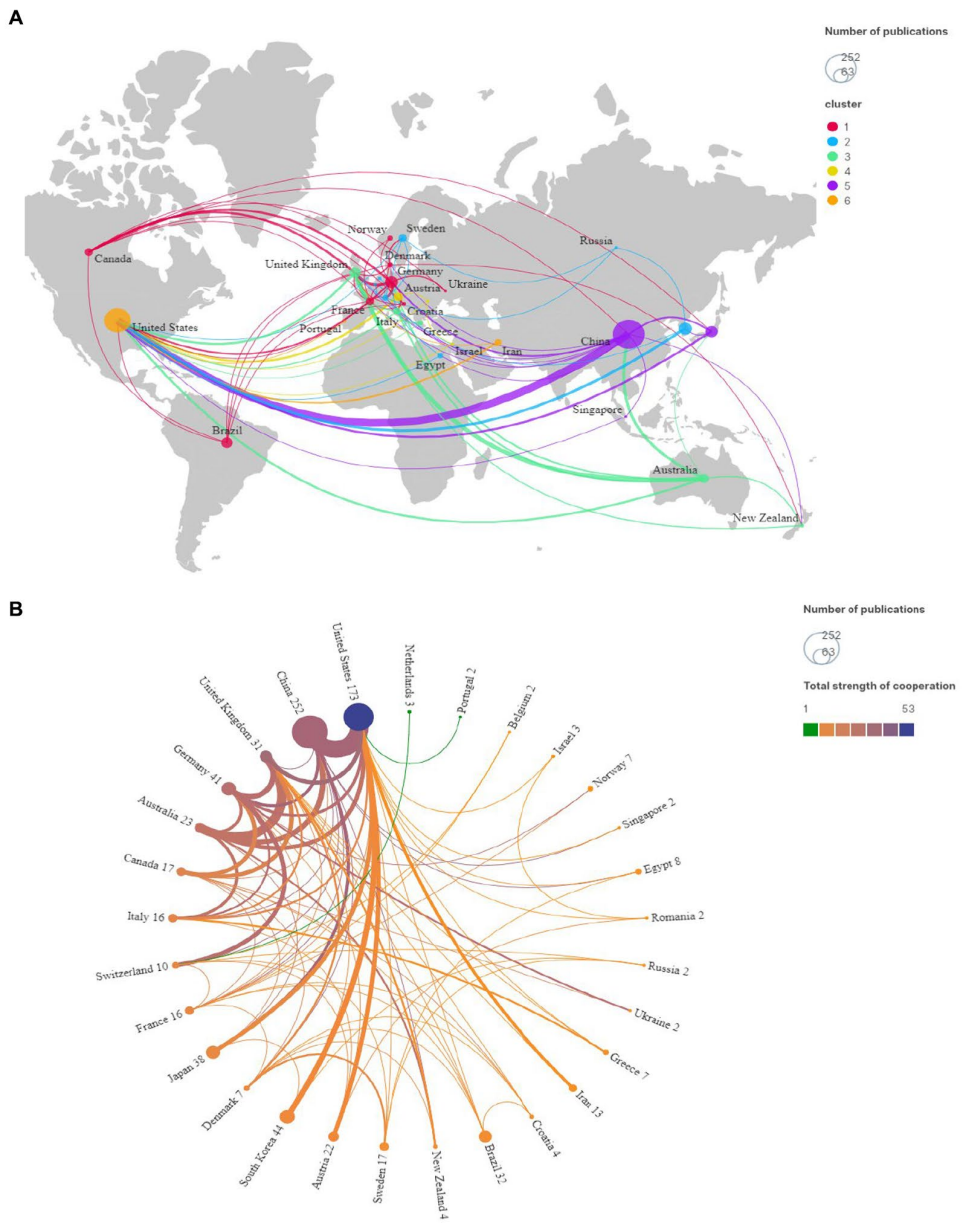
**(A)** Map of the geographic location of countries/regions. **(B)** Chord chart of the strength of countries/regions cooperation.

In total, 746 references were published by 51 countries or regions. To improve visual clarity, this study used VOSviewer and Scimago Graphica to select 30 countries or regions with more than two articles, resulting in 6 clusters and 83 links (Figure 3A). The thickness of the line between countries indicates the level of cooperation (Figure 3B). Co-occurrence map analysis of institutions reveals that China cooperates with a wide range of countries, with 16 having published ten or more papers. China ranked first in the number of publications (252 papers), followed by the United States, South Korea, Germany, Japan, Brazil, and the United Kingdom, etc.

The United States exhibited the highest centrality (0.62), indicating relevance and strong cooperation in this field. China, although the top publisher, had a centrality of only 0.25. This finding highlights that China and the United States play critical roles in research in this field, but academic exchanges between the two countries remain limited. As the birthplace of acupuncture anesthesia, China should seek more international cooperation to promote the global use of acupuncture anesthesia. The top 10 countries/regions and institutions are listed in Table 2.

**Table 2:** Top 10 countries and institutions with the highest frequency and centrality on acupuncture anesthesia.

Rank	Country	Publications	Centrality	Institution	Publications	Centrality
1	China	252	0.25	University of California System	21	0.16
2	United States	173	0.62	Capital Medical University	20	0.15
3	South Korea	44	0.02	Kyung Hee University	16	0.05
4	Germany	41	0.13	China Academy of Chinese Medical Sciences	13	0.14
5	Japan	38	0.02	China Medical University	13	0.08
6	Brazil	32	0.13	Air Force Medical University (the Fourth Military Medical University)	12	0.02
7	United Kingdom	31	0.13	Guangzhou University of Chinese Medicine	12	0.06
8	Australia	23	0.08	Yale University	11	0.01
9	Austria	22	0.06	Chengdu University of Traditional Chinese Medicine	10	0.03
10	Turkey	19	<0.01	Peking University	9	0.06

University of California System (University of California System) includes University of California, Irvine (9 publications), University of California, Los Angeles (6 publications), and other campuses.

**Figure 4:**
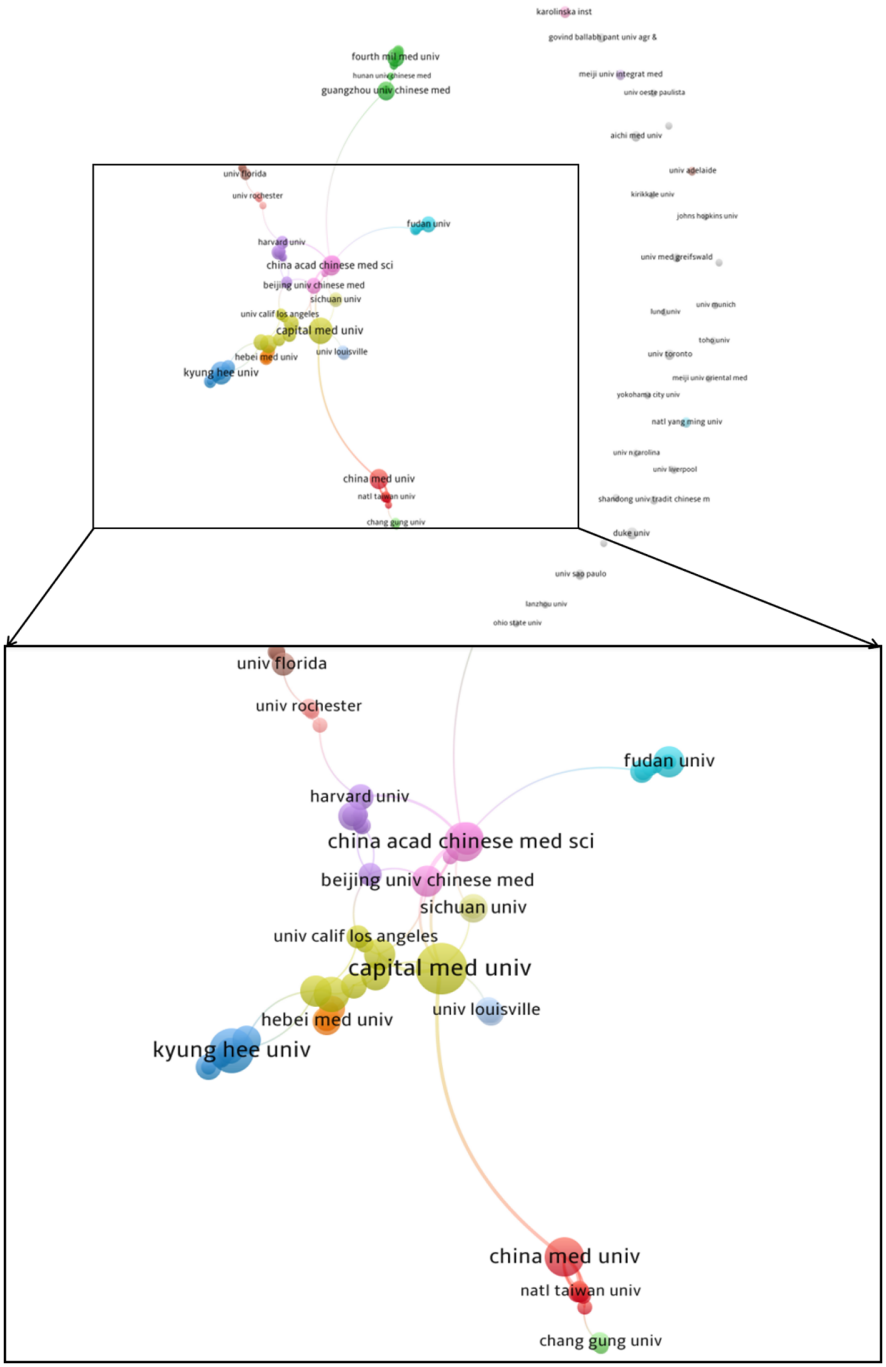
Map of institutions with publications in acupuncture anesthesia.

A total of 953 research institutions were involved, and 114 institutions with more than 3 papers were chosen for visualization. We performed the co-occurrence analysis of institutions using VOSviewer, resulting in 42 clusters and 135 links (Figure 4). Our analysis found a relatively low level of centrality, indicating that collaboration among institutions is not well-established. The top 10 institutions in terms of the number of publications were the University of California System (21), Capital Medical University (20), Kyung Hee University (16), China Academy of Chinese Medical Sciences (13), China Medical University (13), Air Force Medical University/Fourth Military Medical University (12), Guangzhou University of Chinese Medicine (12), Yale University (11), Chengdu University of Traditional Chinese Medicine (10), and Peking University (9). In the future, greater academic exchange and cooperation among universities and research institutions could promote the further development of acupuncture anesthesia.

### 3.4. Analysis of authors and co-cited author

**Figure 5:**
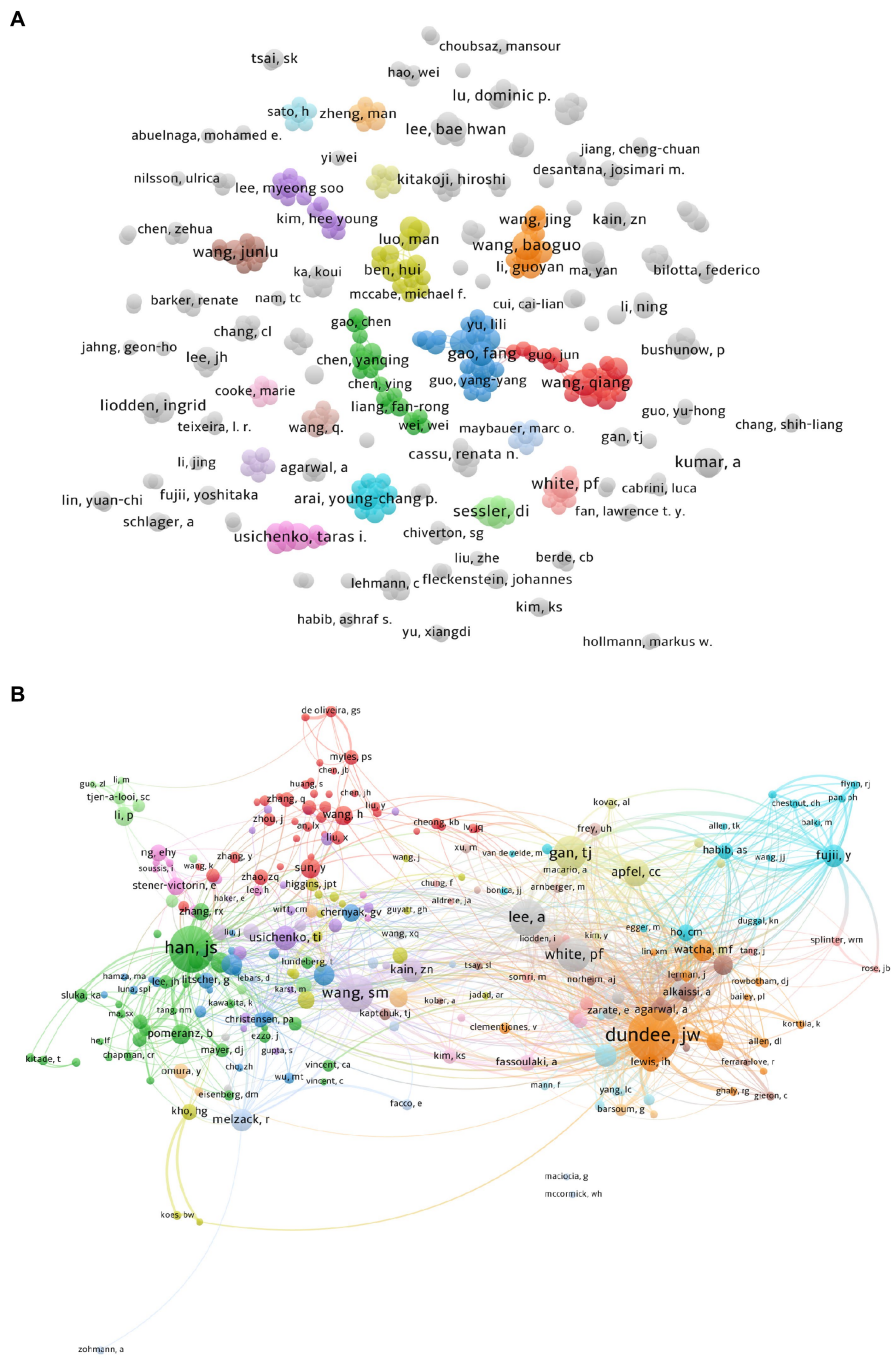
**(A)** Collaboration map of Authors with publications in acupuncture anesthesia. **(B)** Collaboration map of Co-cited Authors with publications in acupuncture anesthesia.

A total of 3,466 authors contributed to these articles; we selected 363 ones with more than two papers for better visualization. We performed the co-occurrence analysis of authors using VOSviewer, resulting in 97 clusters and 687 links (Figure 5A). The relationships between authors can also be observed on this map. In the top 10 authors listed in Table 3. The co-authorship network shows prolific authors and the collaboration among them. The largest number of papers was 7 (Aashish J. Kumar, Daniel I. Sessler, Baoguo Wang and Paul F. White), followed by 6 (Taras I. Usichenko and Qiang Wang). Although many authors have published relevant articles, there was little collaboration among them. Furthermore, the centrality of the authors was relatively low, suggesting that more large-scale, high-quality collaborations are needed in the future.

**Table 3:** Top 10 authors and co-cited authors with the highest frequency in acupuncture anesthesia.

Rank	Author	Publications	Centrality	Co-cited Author	Citations	Centrality
1	Aashish J. Kumar	7	<0.01	DUNDEE, JW	228	0.13
2	Daniel I. Sessler	7	<0.01	HAN, JS	188	0.23
3	Baoguo Wang	7	<0.01	Wang, Shu-Ming	135	0.09
4	Paul F. White	7	<0.01	Lee, Anna	129	0.06
5	Taras I. Usichenko	6	<0.01	Gan, Tong Joo	118	0.06
6	Qiang Wang	6	<0.01	White, Paul F.	111	0.07
7	Fang Gao	5	<0.01	Apfel, CC	82	0.04
8	Bae Hwan Lee	5	<0.01	Usichenko TI	67	0.02
9	Shuqin Li	5	<0.01	Kain, Zeev N	66	<0.01
10	Yanan Li	5	<0.01	Yentis, S. M.	60	0.02

Fifteen thousand nine hundred seventy-seven co-cited authors contributed to these articles; we selected 252 ones with more than 10 papers for better visualization. The Collaboration map of Co-cited Authors consisted of 22 clusters and 10,940 links, and we chose to visualize the largest connected component only (Figure 5B). DUNDEE, JW, from the Department of Anesthetics at Queen’s University of Belfast in Northern Ireland is the author with the highest number of citations (228). As early as 1990, Garwin and his colleagues combined the clinical findings of P6 (Neiguan) stimulation for postoperative sickness with the literature to provide evidence supporting the use of acupuncture for all types of vomiting (34). The top 10 authors and Co-cited authors are listed in Table 3.

### 3.5. Analysis of co-occurrence keywords

**Figure 6:**
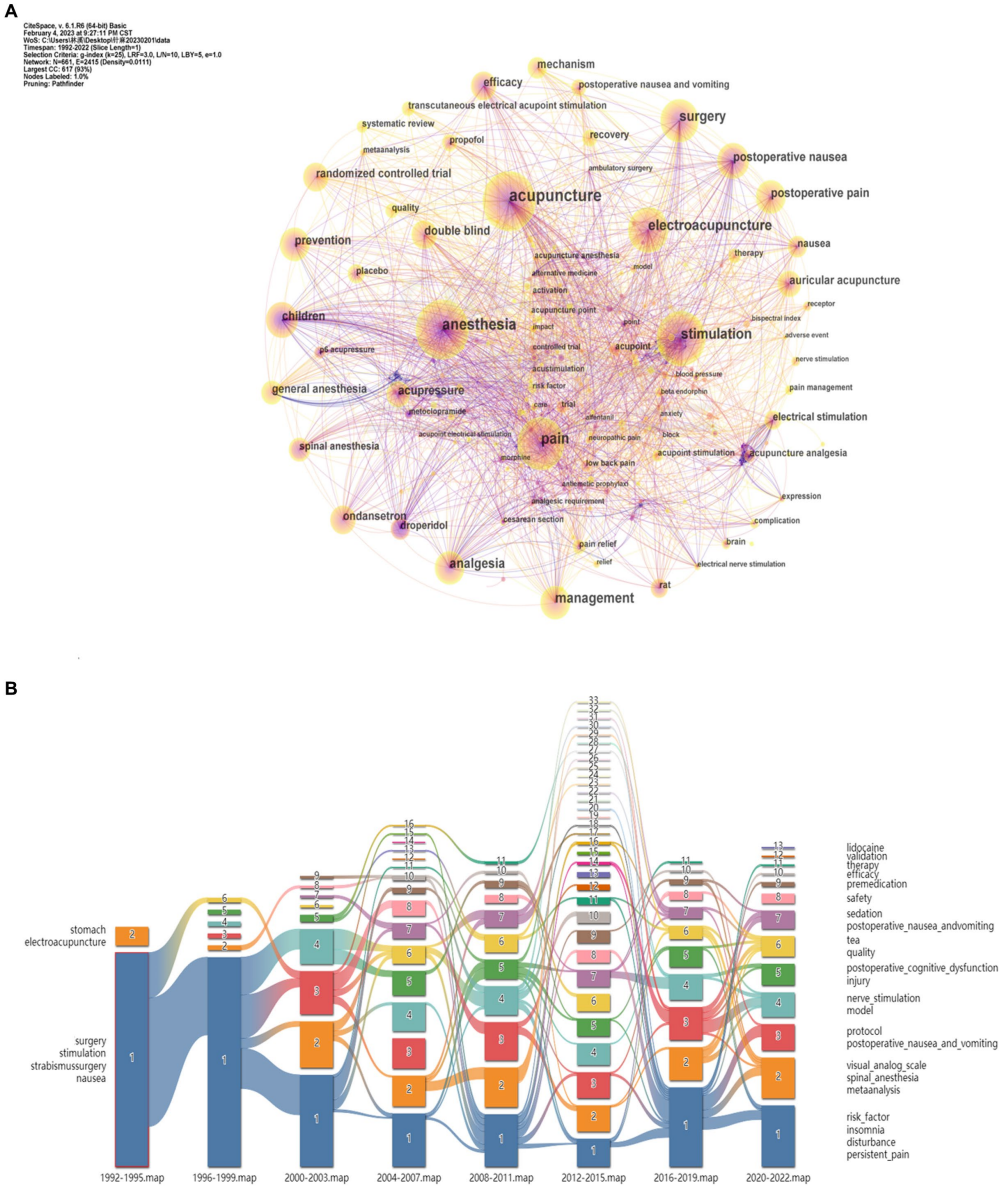
**(A)** Map of keywords with publications in acupuncture anesthesia. **(B)** An alluvial diagram illustrating the emergence of acupuncture anesthesia.

Co-occurrence refers to the phenomenon in which two or more keywords appeared in other literature at the same time. The 746 publications on acupuncture anesthesia brought of 661 keywords together. A total of 661 nodes and 2,415 links (density = 0.0111) comprised the merged co-keyword network. When “Pathfinder” and “pruning sliced networks” were applied, a co-occurrence map of keywords, Figure 6A, was generated. The alluvial diagram of the changes from year to year in keywords for acupuncture anesthesia is shown in Figure 6B. The 20 most frequent co-occurrences refer to the phenomenon in which two or more keywords appear together in the literature. Table 4 lists the 20 most frequently co-occurring keywords. Undoubtedly, “acupuncture” and “anesthesia” were the two most frequent, with 202 and 141 publications, respectively. Keywords related to the retrieval strategy were removed and the top 10 keywords were, in descending order of frequency, pain, electroacupuncture, stimulation, surgery, management, analgesia, postoperative nausea and prevention. “acupuncture” had the highest centrality (0.22), followed by “anesthesia” (0.17) and “pain” (0.16).

**Table 4:** Top 20 keywords with the highest frequency in acupuncture anesthesia.

Rank	Keyword	Frequency	Centrality	Year	Rank	Keyword	Frequency	Centrality	Year
1	Acupuncture	202	0.22	1993	11	Postoperative pain	47	0.08	2002
2	Anesthesia	141	0.17	1993	12	Acupressure	46	0.08	1993
3	Pain	115	0.16	1993	13	Children	45	0.11	1992
4	Electroacupuncture	109	0.08	1999	14	Double blind	45	0.09	2001
5	Stimulation	91	0.15	1993	15	General anesthesia	38	0.06	1992
6	Surgery	83	0.05	1993	16	Auricular acupuncture	38	0.07	2003
7	Management	75	0.07	1994	17	Efficacy	36	0.05	2000
8	Analgesia	68	0.14	1992	18	Mechanism	36	0.06	2010
9	Postoperative nausea	49	0.09	1993	19	Ondansetron	35	0.03	2000
10	Prevention	47	0.04	1993	20	Randomized controlled trial	35	0.06	2006

### 3.6. Analysis of burst keywords

**Figure 7:**
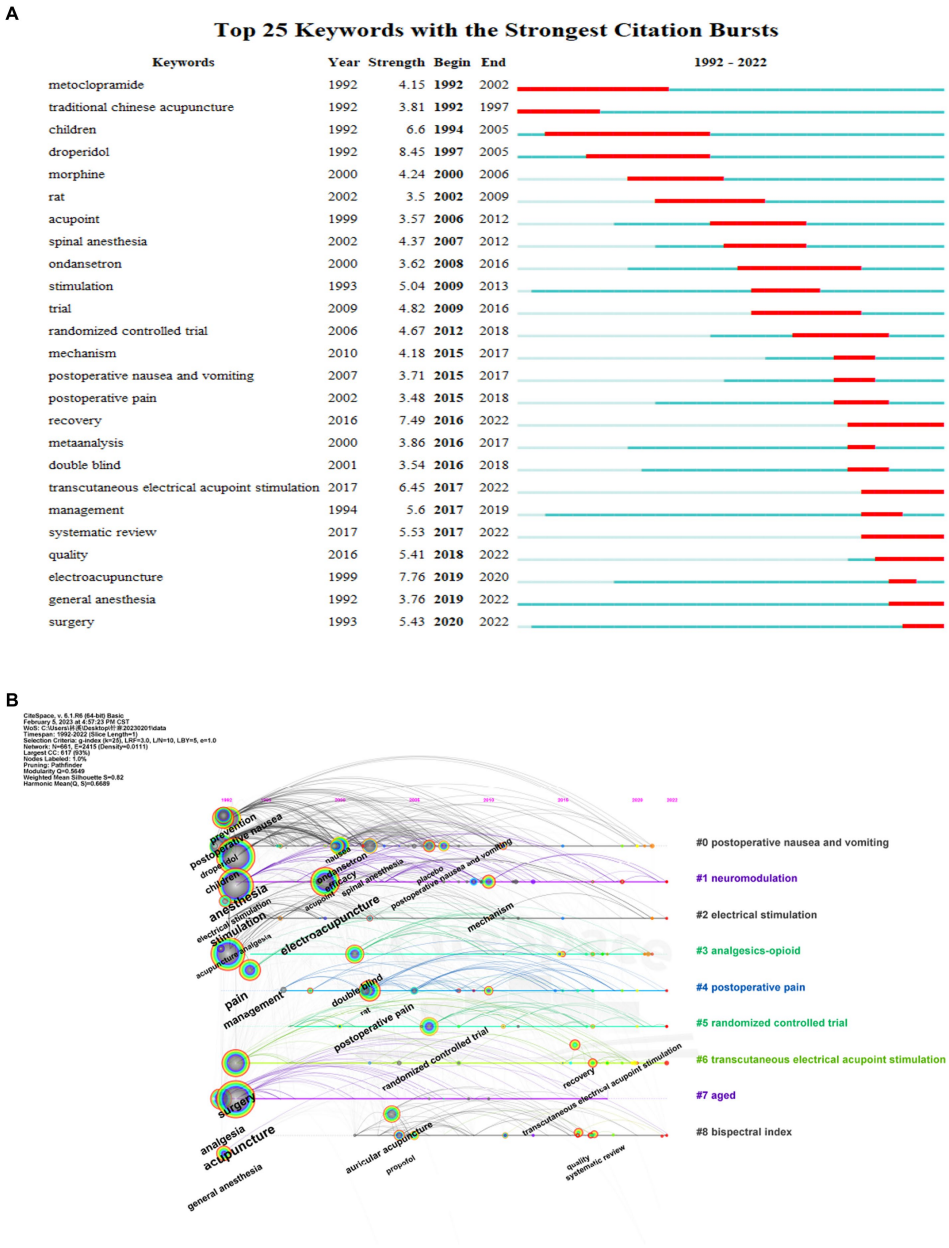
**(A)** Top25 keywords with the Strongest Citation Bursts in acupuncture anesthesia. g-index (*k* = 25). gamma = 1.0. The discontinuous blue lines represent the timeline, specifically, each small blue rectangle represents 1 year, and the red part in the timeline represents the burst duration of the keyword. **(B)** Timeline view of the keywords in acupuncture anesthesia. The top nine clusters were arranged on a horizontal timeline, and the direction of time points to the right from 1992 to 2022. The horizontal lines are timelines, with different color in each cluster. The tree rings represent occurrence of keywords, and the larger rings represent more frequency of occurrence. The camber line above the horizontal line represents the co-occurrence relationship between keywords.

“Burst keywords” refer to keywords cited frequently over some time, thereby indicating the frontier areas. Figure 7 shows twenty burst keywords sorted by the “begin year.” As displayed, the related investigation heat from the keyword “metoclopramide” and “children” has lasted for more than 10 years. And then, “droperidol” became popular among researchers from 1997 to 2005, with the highest strength (8.45) among these 25 burst keywords. Currently, six keywords had become burst and have lasted until now: recovery, transcutaneous electrical acupoint stimulation, systematic review, quality, general anesthesia, and surgery.

### 3.7. Analysis of co-citation references and co-citation journals

It is believed that the citation rate can not only measure the impact or importance of a certain work but also reflect its recognition within the scientific community. Therefore, identifying highly cited papers can help recognize the papers or topics in acupuncture anesthesia that have received the most recent attention from the scientific community. Considering that citations accumulate over time, we present the top 10 highly cited papers during the last decade based on the average citations per year in Table 5.

**Table 5:** Top 10 Co-citation references with the highest frequency in acupuncture anesthesia (within 10 years).

Rank	Representative author (publication year)	Title of reference	Frequency	Centrality	DOI	Journal			Type of research
						Name	IF (2021)	H-index (2021)	
1	Wang et al. (35)	Transcutaneous electric acupoint stimulation reduces intra-operative remifentanil consumption and alleviates postoperative side-effects in patients undergoing sinusotomy: a prospective, randomized, placebo-controlled trial	20	0.04	10.1093/bja/aeu001	British Journal of Anesthesia	11.719	189	RCT
2	Asmussen et al. (10)	Effects of Acupuncture in Anesthesia for Craniotomy: A Meta-Analysis	16	0.08	10.1097/ANA.0000000000000290	Journal of Neurosurgical Anesthesiology	4.01	66	Meta-analysis
3	Gan et al. (36)	Society for Ambulatory Anesthesia. Consensus guidelines for the management of postoperative nausea and vomiting	16	0.08	10.1213/ANE.0000000000000002	Anesthesia and Analgesia	6.627	208	Guideline
4	Wu et al. (37)	The Efficacy of Acupuncture in Post-Operative Pain Management: A Systematic Review and Meta-Analysis	15	0.07	10.1371/journal.pone.0150367	PLoS One	3.752	367	Meta-analysis
5	An et al. (38)	Electro-acupuncture decreases postoperative pain and improves recovery in patients undergoing a supratentorial craniotomy	12	0.07	10.1142/S0192415X14500682	American Journal of Chinese Medicine	6.005	67	RCT
6	Gao et al. (39)	Transcutaneous electrical acupoint stimulation for prevention of postoperative delirium in geriatric patients with silent lacunar infarction: a preliminary study	11	0.01	10.2147/CIA.S183698	Clinical Interventions in Aging	3.829	85	RCT
7	Huang et al. (40)	Effects of transcutaneous electrical acupoint stimulation at different frequencies on perioperative anesthetic dosage, recovery, complications, and prognosis in video-assisted thoracic surgical lobectomy: a randomized, double-blinded, placebo-controlled trial	11	0.07	10.1007/s00540-015-2057-1	Journal of Anesthesia	2.931	49	RCT
8	Lee et al. (41)	Stimulation of the wrist acupuncture point PC6 for preventing postoperative nausea and vomiting	11	< 0.01	10.1002/14651858.CD003281.pub4	Cochrane Database of Systematic Reviews	11.874	292	Meta-analysis
9	Liu et al. (42)	Intraoperative and postoperative anesthetic and analgesic effect of multipoint transcutaneous electrical acupuncture stimulation combined with sufentanil anesthesia in patients undergoing supratentorial craniotomy	11	0.02	10.1136/ACUPMED-2014-010749	Acupuncture in Medicine	1.976	48	RCT
10	Zhang et al. (43)	Mechanisms of Acupuncture-Electroacupuncture on Persistent Pain	10	0.14	10.1097/ALN.0000000000000101	Anesthesiology	9.198	245	Review

RCT: Randomized Controlled Trial.

**Figure 8:**
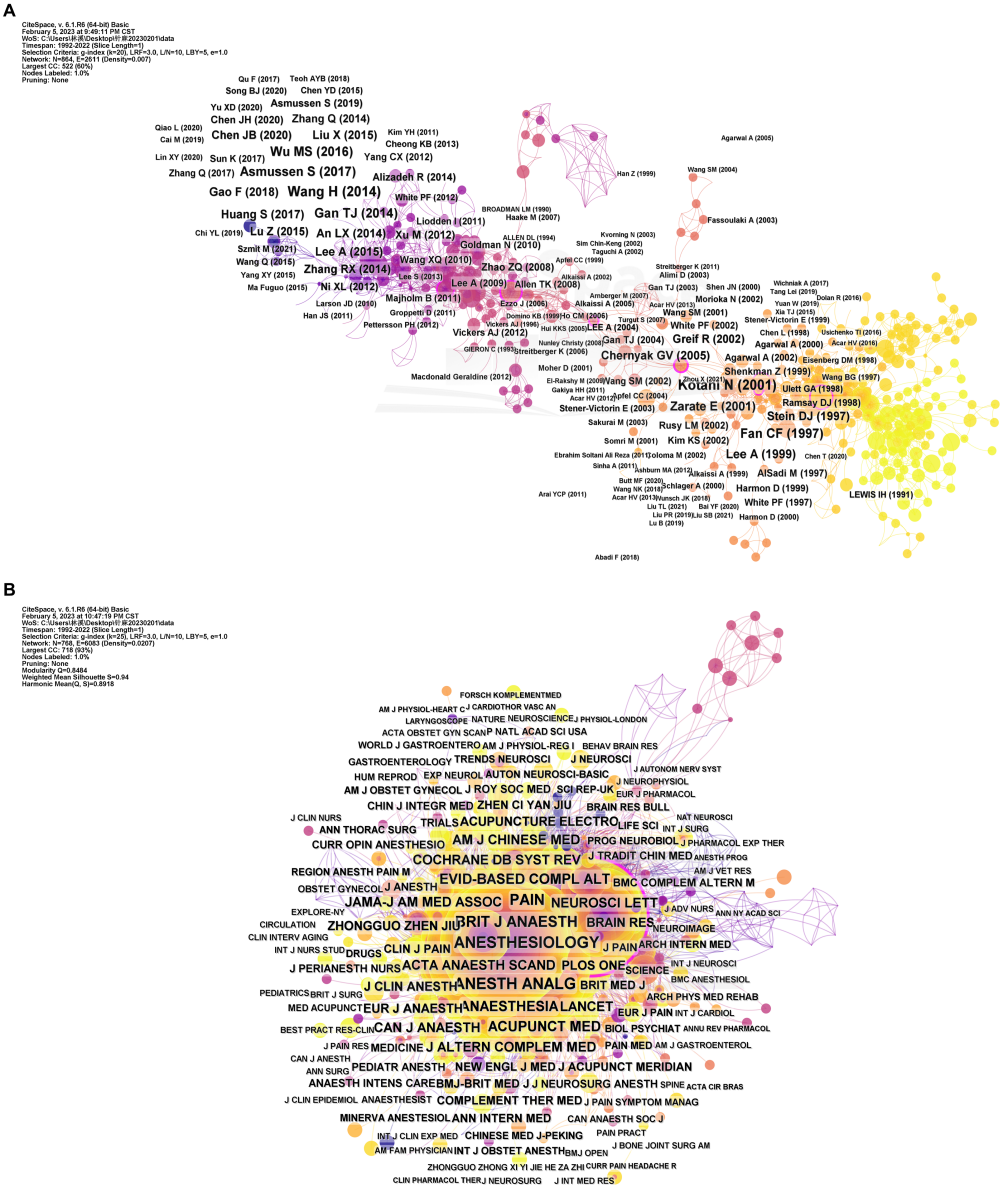
**(A)** Co-occurrence network of co-citation references (threshold = 3). **(B)** Co-occurrence network of co-citation journals (threshold = 15).

A total of 864 nodes and 2,611 links (density = 0.007) comprised the co-citation reference network. A total of 864 references were extracted from the 746 articles on acupuncture anesthesia to analyze the cited references (Figure 8A). The first cited literature was published in 1987, and the most recent was published in 2022. Five of these were RCTs, three meta-analyzes, one review, and one guideline. *Transcutaneous electric acupoint stimulation reduces intra-operative remifentanil consumption and alleviates postoperative side-effects in patients undergoing sinusotomy: a prospective, randomized, placebo-controlled trial* (35) by Wang et al., published in 2014, topped the list with 20 citations during the last decade. Written by Zhang et al., Mechanisms of Acupuncture-Electroacupuncture on Persistent Pain (IF: 9.198), published in 2014 had the highest centrality of 0.25 during the last decade.

A total of 768 nodes and 6,083 links (density = 0.0207) comprise the co-citation journal network (Figure 8B). *Anesthesia and Analgesia* (*Anesth Analg*, IF:6.627) topped the list with 408 citations. *Acupuncture and Electro-Therapeutics Research* (*Acupuncture Electro*, IF: 0.684) had the highest centrality (0.13), with 104 citations. The top 10 co-citation journals listed in Table 6.

**Table 6:** Top 10 Co-citation journals with the highest frequency in acupuncture anesthesia.

Rank	Cited journal	Frequency	Centrality	IF (2021)	H-Index (2021)	Year
1	Anesthesia and Analgesia	408	0.02	6.627	208	1992
2	Anesthesiology	353	0.02	9.198	245	1992
3	British Journal of Anesthesia	337	0.02	11.719	189	1992
4	Pain	266	0.04	7.926	269	1992
5	Anesthesia	232	0.04	12.893	124	1992
6	Acupuncture in Medicine	197	0.07	1.976	48	1996
7	Evidence-Based Complementary and Alternative Medicine	187	0.01	2.65	100	2007
8	Acta Anaesthesiologica Scandinavica	172	0.07	2.274	112	1992
9	Lancet	170	0.06	202.731	807	1992
10	Cochrane Database of Systematic Reviews	158	0.03	11.874	292	2006

## 4. Discussion

The meridian system, known as jingluo, is a fundamental concept in both physiology and pathology. Acupoints, located over these meridians, are selected sites for acupuncture. Acupuncture points refer to specific sites on the body surface where meridians, qi, and blood gather. These sites serve as stimulation points and therapeutic foundations for acupuncture, as well as sensory and reaction points for physiological functions and pathological changes of internal organs. Acupuncture points are related to immune, neurological, and endocrine regulatory networks, and regulating these acupoints can promote overall bodily regulation for achieving balance and maintenance of harmonious internal and external environments (44). When acupuncture stimulation is applied to acupuncture points, it promotes releases special molecules, like opioid peptide, glutamate, and adenosine calcium, which alleviate pain on particular organs (43). Several clinical studies (11, 19, 45, 46) have demonstrated that acupuncture anesthesia can effectively reduce the dosage of anesthetic drugs and the risk of adverse reactions in surgical applications, with minimal physiological intervention in patients. Adenosine, a neuromodulator with antinociceptive characteristics, is involved in the local analgesic effect of acupuncture and amplifies the rise in acupuncture-induced adenosine and its antinociceptive effect (47). Given its clinical significance, the research, discovery, and innovation of acupuncture anesthesia is a crucial and interesting field of study. This research seeks to produce a more comprehensive and thorough analysis of the development of acupuncture anesthesia in the last three decades using bibliometric data.

Currently, Western culture and science and technology have exerted a significant influence on acupuncture anesthesia’s development, resulting in various emerging challenges. However, this phenomenon also represents an opportunity for its growth (48). Notably, a new model of comprehensive perioperative management of acupuncture anesthesia with the characteristics of integrated traditional Chinese and Western medicine has gradually been forming in China (49). To strengthen the practice of acupuncture anesthesia, researchers must promote exchange and cooperation actively. They should absorb and embrace the latest evidence-based concepts, research methods, and techniques to expand the scope of acupuncture anesthesia’s application. This approach should take into account the traditional connotations of this method while also acknowledging its limitations critically. To ensure the continued success of acupuncture anesthesia, researchers must follow the law of cross-disciplinary integration and innovative development. By doing so, they can maintain absorption, collaboration, innovation, and continuously improve the quality, vitality, and development of acupuncture anesthesia.

Our study found that in terms of research methods, RCTs, systematic reviews, and meta-analyzes are the mainstream of evidence-based medicine and are widely used in clinical practice. Research hotspots and directions have expanded from theoretical basis, historical evolution, clinical observation, and safety studies to mechanism studies, perioperative analgesia, and enhanced recovery after surgery (ERAS).

Since its first publication in 1992, the field of acupuncture anesthesia has experienced a fluctuation in growth. However, current trend analysis suggests that there will be a continued growth trend and greater development space in the coming years. One significant breakthrough in acupuncture anesthesia occurred in early 20th century Shanghai, China, where a successful case of mitral valvuloplasty was completed under this method and later televised in the BBC documentary *“Alternative Therapy: Acupuncture”* (1, 50). This event marked the re-entry of acupuncture anesthesia into the global spotlight, allowing it to gain international recognition and attention (1). This boom may have influenced the academic community as well, and the inflection points of steady growth in the field of acupuncture anesthesia also occurred after 2005, with an annual publication volume of more than 20.

The country co-occurrence analysis showed that China, the United States, and South Korea contributed the top three in terms of the number of publications, with the United States having the highest intensity of collaboration. This may be related to the historical origin of the belief that China and the United States have shared a common interest in acupuncture anesthesia since the 1970s. China should take advantage of being the birthplace of acupuncture anesthesia to strengthen international cooperation. Furthermore, collaboration between institutions shows a clear cluster character, with clusters being relatively distant and dispersed, which means that there are currently fewer reciprocal ties between institutions, and no global collaboration has yet been formed.

The co-author network map shows that the centrality of the top 10 authors in terms of number of posts is less than 0.01, reflecting the lack of collaboration among authors and the need for improvement. This highlights the pressing need to strengthen international exchange and collaboration in the field of acupuncture anesthesia. There are numerous international scientific research institutions and teams that possess a significant interest in acupuncture anesthesia and are at the forefront of scientific advancements. Therefore, it is important to expand the avenues of international academic exchanges on acupuncture anesthesia. This could be achieved by strengthening the drive to inherit innovation, encouraging researchers, teachers, and students alike to learn new theories, ideas, technologies, and methods, and carrying out and reinforcing cooperative experiments on acupuncture anesthesia. We could also establish international joint research centers and laboratories for acupuncture anesthesia to provide a high-tech platform for the development of acupuncture anesthesia research and innovation. By integrating the resources and strengths of all relevant parties, strengthening exchanges, and promoting mutual progress, we should aim to improve the stakeholder network for acupuncture anesthesia. Through systematic and orderly guidance, stakeholders can jointly promote the development and innovation of a series of high-quality acupuncture anesthesia research studies.

Keywords such as “pain,” “electroacupuncture,” stimulation,” “surgery,” “analgesia,” and “postoperative nausea” appear early and frequently and were the focus of attention in this field. Since 2006, the emergence of the high-frequency keywords “randomized controlled trial” and “mechanism” suggests that international research has gradually paid attention to quality control and mechanism exploration of studies related to acupuncture anesthesia. In terms of indications, acupuncture anesthesia is being investigated not only for clinical anesthesia management but also for perioperative pain, nausea and vomiting, and other symptoms that can hasten patient recovery (51). In terms of applicable groups, it is being applied to special populations aimed at elderly, children and pregnant women (46, 52, 53). This approach complies with the principles of ERAS (54), an evidence-based, multimodal, and multidisciplinary approach to surgical patient care. ERAS aims to enhance perioperative management and outcomes (55). This is in line with the recommendations of various guidelines, which suggest that opioids should be used sparingly. Reduced opioid use is believed to improve patient outcomes and reduce the risk of addiction and overdose.

The research frontiers were identified by analyzing the burst keywords The five most recent burst keywords were “recovery” “transcutaneous electrical acupoint stimulation” “systematic review” “quality” “general anesthesia” and “surgery.” Facilitating rehabilitation anesthesia management and reduction of opioid abuse has been a topic of interest in surgical departments (56–58). Despite progress in research more efforts are needed to understand the characteristics of the role of acupuncture anesthesia in rehabilitation sedation and mechanisms to reduce anesthetic drugs. In contrast transcutaneous electrical acupoint stimulation is widely used in the perioperative period instead of traditional needling because of its ease of operation and noninvasive nature. Quality indicates that the field of acupuncture anesthesia is placing increasing emphasis on the credibility of research and future acupuncture anesthesia research can be speculated to be of higher quality standardized and institutionalized. Clinical trial design standardization of operational procedures and optimization of the scaling of perioperative effects have become popular (7). For example establish a core outcome set to assessing the effectiveness of acupuncture anesthesia and reach a consensus by integrating the opinions of different stakeholder groups (59).

The keyword timeline view describes the chronological sequence of keywords that appear on the horizontal timeline and displays the dynamic time change of clustering keywords. Combined with the alluvial diagram, it can be seen that the research frontier has variability at different time stages, which is related to various factors, such as national policies, the development of medical research, the social environment, the awareness and acceptance of the patient and the inheritance of acupuncture anesthesia techniques. From 1992 to 2004, the research frontier focused on the advancement of effectiveness and the improvement of postoperative nausea and vomiting in the areas of anesthetic drugs, acupuncture points, electrical stimulation, and effectiveness. From 2005 to 2013, the research frontier migrated to mechanisms, double-blind placebo, conventional anesthesia, and postoperative pain (24, 60). From 2014 to 2022, the research frontier includes systematic evaluation, randomized controlled trials, quality, and recovery, with a greater focus on evidence-based integration of outcome indicators and the promotion of multifaceted and multilevel recovery after surgery. Focusing on evidence-based medicine, the researchers optimized perioperative management by reducing the dose of anesthetic drugs to reduce the physiological stress response of surgical patients and promote their recovery. On this basis, Chinese scholars proposed “*perioperative acupuncture medicine*,” which is the use of acupuncture techniques to optimize preoperative, intraoperative and postoperative treatment (61).

Based on the above results, we believe that acupuncture anesthesia has accumulated certain clinical research and theoretical basis, and the combination of frontier keywords and related research (48) can predict that acupuncture will have better combinations and advantages throughout the perioperative phase. However, advancements in precision medicine have increased the need for precise population stratification (62), and the effects of needling and anesthesia management are influenced by complex individual characteristics, highlighting the need for more precise research. Furthermore, due to the high heterogeneity of surgical procedures, the nature of the surgical intervention is difficult to reveal, and the multidisciplinary collaboration is more conducive to the development of this discipline. As a result, we should promote collaboration across industries, the mutual benefit of sharing scientific research resources, and cultivating professionals skilled in acupuncture, surgery, and anesthesia. Researchers must harness the synergies of multidisciplinary approaches (59) while actively using big data, MRI (63) and positron emission tomography (PET), systems research and machine learning (64) to clarify the specific roles and mechanisms of acupuncture anesthesia in different surgical procedures, in order to inform future development and preventative strategies (65).

### 4.1. Highlights and limitations

To our knowledge, this is the first study to use bibliometric analysis to summarize the progress of acupuncture anesthesia, visually presenting authors, institutional collaboration networks, research hotspots and development prospects (22). Future scholars can read this article to understand the current development situation, identify development prospects, and develop research ideas. We hope to further strengthen cooperation and communication through better understanding of the contributions of scholars and institutions with in this field. This study had some limitations. First, owing to the limitations of CiteSpace data analysis, we only collected literature from the WOS core collection database. We did not include studies from Chinese or other English databases. Some original Chinese studies were excluded, which affected the results. Articles may have different citation counts and centrality when searching for different time periods. Therefore, this study only presents results from the past 31 years. Furthermore, due to the purpose and type of research, the specific mechanism of acupuncture anesthesia has not been definitively determined.

## 5. Conclusion

In this study, we used CiteSpace, VOSviewer, and other visualization software to describe the research progress of acupuncture anesthesia from 1992 to 2022, recent hotspots of concern and exposed problems, provide references to predict future development trends, and make suggestions for reflection. These results indicate a steady increase in international publications related to acupuncture anesthesia after 2005, suggesting a growing research base in this field. With the change in emphasis on the direction of perioperative acupuncture and analgesia, research in this field is developing in a more favorable direction. However, previous studies have faced challenges such as inconsistent theories, unknown mechanisms, and low quality. A core strength of research has yet to develop, and there is a lack of common understanding and cooperation among researchers, as well as a lack of high-quality basic research. Nevertheless, these challenges reflect that research in this field is currently in a transition stage of exploring and integrating disciplinary knowledge more deeply, with a promising future ahead.

## Data availability statement

The original contributions presented in the study are included in the article/Supplementary material, further inquiries can be directed to the corresponding authors.

## Author contributions

KW and JZ conceived and designed and revised manuscripts. LS and XW contributed to data collection, visualization, and manuscript writing. JZ contributed to obtaining funding for the study and project administration. All authors reviewed the manuscript and approved the submitted version.

## Funding

The work was supported by the National Natural Science Fund of China (82074163 and 81973940), Shanghai Clinical Research Center for Acupuncture and Moxibustion (20MC1920500), Science and Technology Commission of Shanghai Municipality (21Y31920100), Clinical Key Specialty Construction Foundation of Shanghai (No. shslczdzk04701), Shanghai Municipal Commission of Health and Family Planning (ZY(2021–2023)-0208), Shanghai Shenkang Hospital Development Center (SHDC2022CRS040), and “Training Plan for Key Talents for Clinical Research” of Affiliated Hospital of Shanghai University of Traditional Chinese Medicine (2023LCRC12).

## Conflict of interest

The authors declare that the research was conducted in the absence of any commercial or financial relationships that could be construed as a potential conflict of interest.

## Publisher’s note

All claims expressed in this article are solely those of the authors and do not necessarily represent those of their affiliated organizations, or those of the publisher, the editors and the reviewers. Any product that may be evaluated in this article, or claim that may be made by its manufacturer, is not guaranteed or endorsed by the publisher.

## Footnotes

^1^https://www.ncbi.nlm.nih.gov/mesh

^2^https://graphica.app/

^3^https://www.mapequation.org/apps/AlluvialGenerator.html

^4^http://www.scimagojr.com/countryrank.php

## References

[1] Zhou J. Historical review about 60 years’ clinical practice of acupuncture anesthesia. Zhen Ci Yan Jiu. (2018) 43:607–10. doi: 10.13702/j.1000-0607.180539 30365253

[2] Jin L Wu J Chen G Zhou L. Unforgettable ups and downs of acupuncture anesthesia in China. World Neurosurg. (2017) 102:623–31. doi: 10.1016/j.wneu.2017.02.036, PMID: 28214637

[3] Cheng TO. Medicine in People's Republic of China. Ann Intern Med. (1973) 78:835. doi: 10.7326/0003-4819-78-5-835_3

[4] Liu L Fan AY Zhou H Hu J. The history of acupuncture anesthesia for pneumonectomy in Shanghai during the 1960s. J Integr Med. (2016) 14:285–90. doi: 10.1016/S2095-4964(16)60253-4, PMID: 27417174

[5] Witte W. Nixon and Scheel in China. Acupuncture and anesthesia in west and East Germany in the 1970s and 1980s. J Anesth Hist. (2018) 4:26. doi: 10.1016/j.janh.2017.11.010 32473763

[6] Huang Y. Documentary research on the spread of acupuncture anaesthesia from China to USA. Chin J Hist Sci Technol. (2009) 30:240–7. doi: 10.3969/j.issn.1673-1441.2009.02.009

[7] Meng Lin X Cheng Lin L Peng F. Progress of the application research of acupuncture anesthesia in thyroid surgery. Zhen Ci Yan Jiu. (2021) 46:168–71. doi: 10.13702/j.1000-0607.200283 33788440

[8] Fleckenstein J Baeumler P Gurschler C Weissenbacher T Annecke T Geisenberger T. Acupuncture reduces the time from extubation to 'ready for discharge' from the post anaesthesia care unit: results from the randomised controlled Acu ARP trial. Sci Rep. (2018) 8:15734. doi: 10.1038/s41598-018-33459-y 30356057 PMC6200780

[9] Li J Fan M Zhou J Zhu YF Gu K Li Q. Coronary arteriography under acupuncture anesthesia: a case report. J Acupunct Tuina Sci. (2018) 16:319–22. doi: 10.1007/s11726-018-1070-y

[10] Asmussen S Maybauer DM Chen JD Fraser JF Toon MH Przkora R. Effects of acupuncture in anesthesia for craniotomy: a Meta-analysis. J Neurosurg Anesth. (2017) 29:219–27. doi: 10.1097/ANA.0000000000000290, PMID: 26967459

[11] Chen J Zhang Y Li X Wan Y Ji X Wang W. Efficacy of transcutaneous electrical acupoint stimulation combined with general anesthesia for sedation and postoperative analgesia in minimally invasive lung cancer surgery: a randomized, double-blind, placebo-controlled trial. Thorac Cancer. (2020) 11:928–34. doi: 10.1111/1759-7714.13343, PMID: 32062864 PMC7113057

[12] Xie M Luo C Feng P. Progress of the application research of acupuncture anesthesia in thyroid surgery. Zhen Ci Yan Jiu. (2021) 46:168–71. doi: 10.13702/j.1000-0607.200283 33788440

[13] Liu Z Wang L Mao H. Clinical effect of acupuncture anesthesia in abortion for first-time pregnancy. Inner Mongolia J Tradit Chin Med. (2013) 32:67. doi: 10.16040/j.cnki.cn15-1101.2013.22.032

[14] Li H Fan M Zhang X. The application of acupuncture anesthesia combined with low dose spinal anesthesia in descending caesarean section. Chin Med Mod Dist Educ Chin. (2019) 17:121–2. doi: 10.3969/j.issn.1672-2779.2019.03.050

[15] Yang Q Qu M Yao Z Zhang X Li C Li L. Comparison of anesthetic effects of acupuncture combined with basic anesthesia and simple basic anesthesia for peritoneal dialysis catheterization. J Hebei Med Univ. (2019) 40:933–6. doi: 10.3969/j.issn.1007-3205.2019.08.016

[16] Gao H Sun X Zhang S Guo X Xu L Guo F. Analysis of transcutaneous electrical acupoint stimulation therapy in gastroscopy. World Latest Med Inform. (2021) 21:135–7. doi: 10.3969/j.issn.1671-3141.2021.87.038

[17] Baum E. Acupuncture anesthesia on American bodies: communism, race, and the cold war in the making of “legitimate” medical science. Bull Hist Med. (2021) 95:497–527. doi: 10.1353/bhm.2021.0055, PMID: 35125353

[18] Lee A Chan S. Acupuncture and anaesthesia. Best Pract Res Clin Anaesthesiol. (2006) 20:303–14. doi: 10.1016/j.bpa.2005.10.009 16850779

[19] Cui H Wu F Wang WT Qian J Li J Fan M. Acupuncture anesthesia for radiofrequency catheter ablation in treatment of persistent atrial fibrillation: a case report. Chin J Integr Med. (2021) 27:137–40. doi: 10.1007/s11655-020-3436-5, PMID: 33140206

[20] Wang P Lu X Wan S Liu C Wang Z. Content and method of literature research on acupuncture anesthesia. J Anhui Tradit Chin Med Coll. (2013) 32:52–4. doi: 10.3969/j.issn.1000-2219.2013.05.017

[21] Chen C. CiteSpace II: detecting and visualizing emerging trends and transient patterns in scientific literature. J Am Soc Inf Sci Technol. (2006) 57:359–77. doi: 10.1002/asi.20317

[22] Belter CW. Bibliometric indicators: opportunities and limits. J Med Libr Assoc. (2015) 103:219–21. doi: 10.3163/1536-5050.103.4.014, PMID: 26512227 PMC4613388

[23] Li X Yin Z Ling F Zheng Q Li X Qi W. The application of acupuncture in cardiopathy: a bibliometric analysis based on web of science across ten recent years. Front Cardiovasc Med. (2022) 9:920491. doi: 10.3389/fcvm.2022.920491, PMID: 36148057 PMC9485815

[24] Liu Y Huang L Xu G Tian H Zhou Z Huang F. The application of acupuncture therapy for postoperative pain over the past 20 years: a bibliometric analysis. J Pain Res. (2022) 15:2085–104. doi: 10.2147/JPR.S371399, PMID: 35923845 PMC9343020

[25] Zhou R Xiao L Xiao W Yi Y Wen H Wang H. Bibliometric review of 1992–2022 publications on acupuncture for cognitive impairment. Front Neurol. (2022) 13:1006830. doi: 10.3389/fneur.2022.1006830, PMID: 36226080 PMC9549373

[26] Zhu S Liu Y Gu Z Zhao Y. A bibliometric analysis ofAdvanced healthcare materials: research trends of biomaterials in healthcare application. Adv Healthc Mater. (2021) 10:2002222. doi: 10.1002/adhm.202002222 33599117

[27] Stout NL Alfano CM Belter CW Nitkin R Cernich A Lohmann Siegel K. A bibliometric analysis of the landscape of Cancer rehabilitation research (1992–2016). J Natl Cancer Inst. (2018) 110:815–24. doi: 10.1093/jnci/djy108, PMID: 29982543 PMC6279275

[28] Dong R Wang H Ye J Wang M Bi Y. Publication trends for Alzheimer's disease worldwide and in China: a 30-year bibliometric analysis. Front Hum Neurosci. (2019) 13:e15718. doi: 10.3389/fnhum.2019.00259 PMC6696880 31447661

[29] Xu D Wang YL Wang KT Wang Y Dong XR Tang J. A Scientometrics analysis and visualization of depressive disorder. Curr Neuropharmacol. (2021) 19:766–86. doi: 10.2174/1570159X18666200905151333, PMID: 32888272 PMC8686305

[30] Sehmbi H Retter S Shah UJ Nguyen S Martin J Uppal V. Epidemiological, methodological, and statistical characteristics of network meta-analysis in anaesthesia: a systematic review. Br J Anaesth. (2022) 130:272–86. doi: 10.1016/j.bja.2022.08.042 36404140

[31] Luo J Shi Y Wang X Zhang R Chen S Yu W. A 20-year Research Trend analysis of the influence of anesthesia on tumor prognosis using bibliometric methods. Front Oncol. (2021) 11:683232. doi: 10.3389/fonc.2021.683232, PMID: 34458138 PMC8397496

[32] Chaomei C. Searching for intellectual turning points: progressive knowledge domain visualization. Proc Natl Acad Sci U S A. (2004) 101:5303–10. doi: 10.1073/pnas.0307513100 14724295 PMC387312

[33] Chen C. Expert review. Science mapping: a systematic review of the literature. J Data Inf Sci. (2017) 2:1–40. doi: 10.1515/jdis-2017-0006

[34] Dundee JW McMillan CM. Clinical uses of P6 acupuncture antiemesis. Acupunct Electrother Res. (1990) 15:211–5. doi: 10.3727/036012990816358153, PMID: 1982043

[35] Wang H Xie Y Zhang Q Xu N Zhong H Dong H. Transcutaneous electric acupoint stimulation reduces intra-operative remifentanil consumption and alleviates postoperative side-effects in patients undergoing sinusotomy: a prospective, randomized, placebo-controlled trial. Br J Anaesth. (2014) 112:1075–82. doi: 10.1093/bja/aeu001, PMID: 24576720

[36] Gan TJ Diemunsch P Habib AS Kovac A Kranke P Meyer TA. Consensus guidelines for the Management of Postoperative Nausea and Vomiting. Anesth Analg. (2014) 118:85–113. doi: 10.1213/ANE.0000000000000002 24356162

[37] Wu M Chen KH Chen IF Huang SK Tzeng PC Yeh ML. The efficacy of acupuncture in post-operative pain management: a systematic review and Meta-analysis. PLoS One. (2016) 11:e150367:e0150367. doi: 10.1371/journal.pone.0150367, PMID: 26959661 PMC4784927

[38] An L Chen X Ren X Wu H. Electro-acupuncture decreases postoperative pain and improves recovery in patients undergoing a Supratentorial craniotomy. Am J Chin Med. (2014) 42:1099–109. doi: 10.1142/S0192415X14500682, PMID: 25169910

[39] Gao F Zhang Q Li Y Tai Y Xin X Wang X. Transcutaneous electrical acupoint stimulation for prevention of postoperative delirium in geriatric patients with silent lacunar infarction: a preliminary study. Clin Interv Aging. (2018) 13:2127–34. doi: 10.2147/CIA.S183698 30425466 PMC6205526

[40] Huang S Peng W Tian X Liang H Jia Z Lo T. Effects of transcutaneous electrical acupoint stimulation at different frequencies on perioperative anesthetic dosage, recovery, complications, and prognosis in video-assisted thoracic surgical lobectomy: a randomized, double-blinded, placebo-controlled trial. J Anesth. (2017) 31:58–65. doi: 10.1007/s00540-015-2057-1 26350110

[41] Lee A Chan SK Fan LT. Stimulation of the wrist acupuncture point PC6 for preventing postoperative nausea and vomiting. Cochrane Database Syst Rev. (2016) 2016:CD003281. doi: 10.1002/14651858.CD003281.pub4, PMID: 26522652 PMC4679372

[42] Liu X Li S Wang B An L Ren X Wu H. Intraoperative and postoperative anaesthetic and analgesic effect of multipoint transcutaneous electrical acupuncture stimulation combined with sufentanil anaesthesia in patients undergoing supratentorial craniotomy. Acupunct Med. (2015) 33:270–6. doi: 10.1136/acupmed-2014-010749, PMID: 25926298

[43] Zhang R Lao L Ren K Berman BM. Mechanisms of acupuncture–electroacupuncture on persistent pain. Anesthesiology. (2014) 120:482–503. doi: 10.1097/ALN.0000000000000101, PMID: 24322588 PMC3947586

[44] Wang B. Application of acupuncture anesthesia in neck surgery. Shanghai J Acumox. (2009) 28:237–8.

[45] Tu Q Yang Z Gan J Zhang J Que B Song Q. Transcutaneous electrical Acupoint stimulation improves immunological function during the perioperative period in patients with non-small cell lung Cancer undergoing video-assisted thoracic surgical lobectomy. Technol Cancer Res Treat. (2018) 17:153303381880647. doi: 10.1177/1533033818806477 PMC6259054 30381011

[46] Xu J Li P Zheng L Chen Q. Effect observation of Electro-acupuncture anesthesia combined with general anesthesia in elderly patients undergoing gastrointestinal tumor resection. Front Surg. (2022) 9:901638. doi: 10.3389/fsurg.2022.901638 35647012 PMC9134448

[47] Goldman N Chen M Fujita T Xu Q Peng W Liu W. Adenosine A1 receptors mediate local anti-nociceptive effects of acupuncture. Nat Neurosci. (2010) 13:883–8. doi: 10.1038/nn.2562, PMID: 20512135 PMC3467968

[48] Yuan W Wang Q. Perioperative acupuncture medicine: a novel concept instead of acupuncture anesthesia. Chin Med J. (2019) 132:707–15. doi: 10.1097/CM9.0000000000000123 30855351 PMC6416101

[49] Jun Jie G Liang Z Jian M. Research progress of acupuncture in enhanced recovery after surgery. Zhen Ci Yan Jiu. (2021) 46:248–53. doi: 10.13702/j.1000-0607.200423 33798300

[50] Zhao H. An analysis of the international communication strategy of Chinese medicine culture in the context of “soft power”[J]. *Southern*. (2017):74–6. doi: 10.3969/j.issn.1004-1133.2017.10.025

[51] Ljungqvist O De Boer HD. Will acupuncture be the next addition to enhanced recovery after surgery protocols? JAMA Surg. (2022) 158:28. doi: 10.1001/jamasurg.2022.5683 36322074

[52] Shao S Chen C Yu Y. Clinical analysis of 557 cases of acupuncture anesthesia in pediatric acute abdomen. Wuhan New Med. (1979):76–8.

[53] Liu X Wu L Yi W. Research progress of acupuncture analgesia for labor. J Acupunct Tuina Sci. (2013) 11:57–60. doi: 10.1007/s11726-013-0656-7

[54] Echeverria-Villalobos M Stoicea N Todeschini AB Fiorda-Diaz J Uribe AA Weaver T. Enhanced recovery after surgery (ERAS). Clin J Pain. (2020) 36:219–26. doi: 10.1097/AJP.0000000000000792 31868759

[55] Kehlet H. Enhanced recovery after surgery (ERAS): good for now, but what about the future? Can J Anesth. (2015) 62:99–104. doi: 10.1007/s12630-014-0261-3, PMID: 25391731

[56] Nicholson A Lowe MC Parker J Lewis SR Alderson P Smith AF. Systematic review and meta-analysis of enhanced recovery programmes in surgical patients. Br J Surg. (2014) 101:172–88. doi: 10.1002/bjs.9394 24469618

[57] Slater RR Beverley L. The opioid epidemic in America: pandemic impacts. J Am Acad Orthop Surg. (2022) 30:e1302:–e1310. doi: 10.5435/JAAOS-D-21-01158, PMID: 35944546

[58] Zhao H Han Q Shi C Feng Y. The effect of opioid-sparing anesthesia regimen on short-term cognitive function after thoracoscopic surgery: a prospective cohort study. Perioper Med. (2022) 11:45. doi: 10.1186/s13741-022-00278-9 PMC9380394 35971162

[59] Zhang Y Jiao RM Witt CM Lao L Liu JP Thabane L. How to design high quality acupuncture trials–a consensus informed by evidence. BMJ. (2022) 376:e67476. doi: 10.1136/bmj-2021-067476 PMC8965655 35354583

[60] Ghai B Jafra A Bhatia N Chanana N Bansal D Mehta V. Opioid sparing strategies for perioperative pain management other than regional anaesthesia: a narrative review. J Anaesth Clin Pharm. (2022) 38:3–10. doi: 10.4103/joacp.JOACP_362_19 PMC9191794 35706649

[61] Zhang W Zhang H Wang SM Guo J Ma Y Li Y. Perioperative acupuncture optimizes surgical outcomes: theory, clinical practice and future perspectives. Am J Chin Med. (2022) 50:961–78. doi: 10.1142/S0192415X22500392, PMID: 35729088

[62] König IR Fuchs O Hansen G von Mutius E Kopp MV. What is precision medicine? Eur Respir J. (2017) 50:1700391. doi: 10.1183/13993003.00391-2017 29051268

[63] Tu C Lee YC Chen YY Chen CM Lu WC Chen YH. Acupuncture treatment associated with functional connectivity changes in primary dysmenorrhea: a resting state fMRI study. J Clin Med. (2021) 10:4731. doi: 10.3390/jcm10204731, PMID: 34682857 PMC8537009

[64] Rashidi P Edwards DA Tighe PJ. Primer on machine learning. Curr Opin Anaesthesiol. (2019) 32:653–60. doi: 10.1097/ACO.0000000000000779, PMID: 31408024 PMC6785021

[65] Tu Y Cao J Bi Y Hu L. Magnetic resonance imaging for chronic pain: diagnosis, manipulation, and biomarkers. Sci China Life Sci. (2021) 64:879–96. doi: 10.1007/s11427-020-1822-4, PMID: 33247802

