# Supplementary material for: Research trends from 1992 to 2022 of acupuncture anesthesia: a bibliometric analysis
Source: Front Med (Lausanne). 2023 Jun 8;10:1194005. doi: 10.3389/fmed.2023.1194005 (PMC10285535; doi:10.3389/fmed.2023.1194005)
Supplement: Supplementary file 1 [file Data_Sheet_1.doc]

Supplementary material. TABLE 1 Ten most-cited papers

| Rank | Representative Author (Publication Year) | Title | Citations | Links | DOI | Journal | IF (Q) | Type of Research | Main finding |
| --- | --- | --- | --- | --- | --- | --- | --- | --- | --- |
| 1 | Watcha M F (1992) | Postoperative nausea and vomiting - its etiology, treatment, and prevention | 1158 | 39 | 10.1097/00000542-199207000-00023 | ANESTHESIOLOGY | 8.986 (1) | review | The causes and therapeutic preventive measures related to postoperative nausea and vomiting are reviewed, and non-pharmacological treatments of acupuncture and acupressure are proposed. |
| 2 | Wu M T (1999) | Central nervous pathway for acupuncture stimulation: Localization of processing with functional MR imaging of the brain - Preliminary experience | 302 | 15 | 10.1148/radiology.212.1.r99jl04133 | RADIOLOGY | 29.146 (1) | Clinical Trial | Acupuncture at ST.36 and LI.4 activates structures of descending antinociceptive pathway and deactivates multiple limbic areas subserving pain association. |
| 3 | Vickers A J (1996) | Can Acupuncture have Specific Effects on Health? A Systematic Review of Acupuncture Antiemesis Trials | 208 | 32 | 10.1177/014107689608900602 | JOURNAL OF THE ROYAL SOCIETY OF MEDICINE | 18.000 (1) | review | Systematic evaluation of 33 controlled trials of acupuncture P6 points for nausea/vomiting associated with chemotherapy, pregnancy, or surgery.Eleven of these trials, involving nearly 2000 patients, showed an effect of P6. |
| 4 | Lee A (1999) | The use of nonpharmacologic techniques to prevent postoperative nausea and vomiting: A meta-analysis | 189 | 54 | 10.1097/00000539-199906000-00031 | ANESTHESIA AND ANALGESIA | 6.627 (2) | meta-analysis | Acupuncture related therapies were equivalent to commonly used antiemetic drugs in preventing vomiting after surgery. Acupuncture related therapies were more effective than placebo in preventing nausea and vomiting within 6 h of surgery in adults, but there was no benefit in children. |
| 5 | Kotani N (2001) | Preoperative intradermal acupuncture reduces postoperative pain, nausea and vomiting, analgesic requirement, and sympathoadrenal responses | 173 | 59 | 10.1097/00000542-200108000-00015 | ANESTHESIOLOGY | 8.986 (1) | RCT | Preoperative insertion of intradermal needles reduces postoperative pain, the analgesic requirement, and opioid-related side effects after both upper and lower abdominal surgery. Acupuncture analgesia also reduces the activation of the sympathoadrenal system |
| 6 | Tan M J (2015b) | Optimizing pain management to facilitate Enhanced Recovery After Surgery pathways | 164 | 3 | 10.1007/s12630-014-0275-x | CANADIAN JOURNAL OF ANESTHESIA-JOURNAL CANADIEN D ANESTHESIE | 6.713 (1) | review | To describe multimodal analgesic techniques, including transcutaneous electrical nerve stimulation, to optimize postoperative pain control and to summarize the evidence for their use in reducing opioid requirements and side effects. |
| 7 | Lin J G (2002) | The effect of high and low frequency electroacupuncture in pain after lower abdominal surgery | 161 | 31 | 10.1016/S0304-3959(02)00261-0 | PAIN | 7.926 (1) | RCT | preoperative treatment with low-EA and high-EA can reduce postoperative analgesic requirements and associated side effects in patients undergoing lower abdominal surgery. |
| 8 | Lee J H (1993) | The distribution of brain-stem and spinal-cord nuclei associated with different frequencies of electroacupuncture analgesia | 145 | 8 | 10.1016/0304-3959(93)90109-3 | PAIN | 7.926 (1) | Animal experiments | Several distinct brain-stem nuclei that may play a role in electroacupuncture-mediated analgesia. |
| 9 | Chen L(1998) | The effect of location of transcutaneous electrical nerve stimulation on postoperative opioid analgesic requirement: Acupoint versus nonacupoint stimulation | 132 | 27 | 10.1097/00000539-199811000-00028 | ANESTHESIA AND ANALGESIA | 6.627 (2) | RCT | Transcutaneous electrical nerve stimulation applied at the dermatomal level of the skin incision is as effective as Zusanli acupoint stimulation, and both were more effective than stimulation at a nonacupoint (shoulder) location. |
| 10 | Lee A (2015) | Stimulation of the wrist acupuncture point P6 for preventing postoperative nausea and vomiting | 127 | 77 | 10.1002/14651858.CD003281.pub4 | COCHRANE DATABASE OF SYSTEMATIC REVIEWS | 12.008 (1) | review | There is low-quality evidence supporting the use of PC6 acupoint stimulation over sham. We found that there is moderate-quality evidence showing no difference between PC6 acupoint stimulation and antiemetic drugs to prevent PONV. Further PC6 acupoint stimulation versus antiemetic trials are futile in showing a significant difference. |
